# DAST-28 severity and injection-related harm risks among people using psychoactive substances in psychiatric and correctional settings in Mongolia: a multicenter cross-sectional study

**DOI:** 10.1016/j.abrep.2026.100736

**Published:** 2026-08-20

**Authors:** Bilguun Nyamdorj, Khongorzul Davaadorj, Tergel Khuyag, Uranchimeg Mungunkhuyag, Munkhnaran Mandakh, Davaalkham Jagdagsuren, Nursabi Khizatkhan, Khishigsuren Zuunnast, Dariimaa Ganbat, Oyunsuren Davaasuren

**Affiliations:** a Department of Mental Health, School of Medicine, Mongolian National University of Medical Sciences, Ulaanbaatar 13320, Mongolia; b National Center for Mental Health, Ulaanbaatar 13320, Mongolia; c National Center for Communicable Diseases, Ulaanbaatar 13335, Mongolia; d Graduate School of Medical Science, Mongolian National University of Medical Sciences, Ulaanbaatar 14210, Mongolia

**Keywords:** DAST-28, Polysubstance use, Injection drug use, Harm reduction, Psychiatric services, Correctional settings, Mongolia

## Abstract

**Introduction:**

Evidence on drug-related problem severity and injection-related harm among people using psychoactive substances in Mongolia is limited, particularly in institutional settings. We examined DAST-28 scores, internal consistency, and behavioral correlates in psychiatric and correctional settings.

**Methods:**

This multicenter cross-sectional study included 181 participants recruited from the National Center for Mental Health and correctional facilities. The continuous DAST-28 total score was the primary outcome. Internal consistency was evaluated from 28 item responses. A clinically specified seven-covariate linear model with HC3 heteroskedasticity-robust standard errors was primary; ordinal and study-defined DAST-28 ≥ 12 models were supportive analyses.

**Results:**

DAST-28 scores were available for 179 participants (median 11.0, interquartile range 6.0–16.0). Among 170 participants with complete item responses, Cronbach's alpha was 0.882 (95% bootstrap confidence interval 0.854–0.902). In the primary model, correctional setting was associated with a 1.82-point lower score (95% CI -3.36 to −0.28), whereas each additional year of use (B = 0.27, 95% CI 0.08–0.45), polysubstance use (B = 2.64, 95% CI 0.79–4.50), and any syringe use (B = 4.43, 95% CI 2.58–6.28) were associated with higher scores. Ordinal and threshold analyses were concordant; variance inflation factors ranged from 1.05 to 1.59.

**Conclusions:**

Greater DAST-28 scores were associated with polysubstance use, syringe use, and longer use duration in these institutional samples. The findings may inform comprehensive assessment and harm-reduction linkage but do not establish a diagnostic cutoff, causal relationship, intervention effectiveness, or population prevalence.

## Highlights

• Continuous DAST-28 scores replaced an unvalidated primary cutoff.
• DAST-28 showed good internal consistency in the institutional sample.
• Syringe use was associated with 4.43-point higher adjusted scores.
• Polysubstance use and longer use duration marked greater severity.
• Findings were consistent across continuous, ordinal, and threshold models.

## 1 Introduction

Global drug markets are changing rapidly, with increasing availability of synthetic substances, diverted pharmaceuticals, new psychoactive substances, and stimulant-type drugs (Peacock et al., 2018; United Nations Office on Drugs and Crime, 2026). Polysubstance use and high-risk routes of administration are associated with complex addiction presentations, psychiatric morbidity, overdose, infectious disease transmission, and premature mortality. People who inject drugs face elevated risks of HIV, hepatitis B virus, hepatitis C virus, skin and soft-tissue infection, and overdose. Comprehensive responses include sterile syringe access, opioid agonist treatment where indicated, overdose prevention, blood-borne virus testing and treatment, and low-threshold engagement with health services (World Health Organization, 2026; World Health Organization, United Nations Office on Drugs and Crime, and UNAIDS, 2012).

Custodial and psychiatric populations may have overlapping but distinct risk profiles. Prisoners and detainees have a high burden of substance-use disorders and blood-borne infections (Dolan et al., 2016; Fazel et al., 2017; Stone et al., 2018), whereas psychiatric services may encounter people during acute clinical crises or periods of severe comorbidity (Drake et al., 2004). In Mongolia, published evidence on drug-related problem severity, gabapentin misuse, synthetic cannabinoid use, and injection-related harm among people already in contact with state institutions remains sparse.

The Drug Abuse Screening Test (DAST) is a self-report measure designed to quantify problems and consequences related to drug misuse; the original version combines 28 binary items into a total score (Skinner, 1982; Yudko et al., 2007). Although DAST versions and cutoffs have been evaluated in specific populations (Cocco & Carey, 1998; Gavin et al., 1989), a locally validated DAST-28 diagnostic threshold is not available for Mongolia. Treating an unvalidated cutoff as the principal outcome may obscure information contained in the full score distribution. We therefore treated the continuous DAST-28 score as primary, ordered score bands as supportive, and the originally used ≥12 threshold only as a sensitivity analysis.

This study aimed to characterize DAST-28 scores and internal consistency; compare psychiatric and correctional participants; and examine associations of demographic characteristics, duration of use, polysubstance use, and syringe use with drug-related problem severity.

## 2 Materials and methods

### 2.1 Study design and reporting

This was a multicenter cross-sectional study of participants with recent psychoactive substance use recruited from psychiatric and correctional settings in Mongolia. Recruitment began after ethics approval in June 2025 and continued through June 2026. Reporting followed the STROBE guidance for cross-sectional studies (von Elm et al., 2007).

### 2.2 Setting and participants

Participants were recruited from the National Center for Mental Health (NCMH), representing psychiatric or addiction-related care, and correctional facilities under the General Executive Agency of Court Decisions (GEACD), representing correctional-system contact related to drug use or drug-related offences. All 181 eligible records available during the study period were included in the analysis-ready dataset: 76 from NCMH and 105 from correctional facilities.

Eligible participants reported psychoactive substance use other than exclusive tobacco or alcohol use and had capacity to complete the interview. The approved protocol permitted inclusion of participants aged <18 years with required guardian consent and participant assent. Two participants were aged 17 years and were retained in the main analysis; a sensitivity analysis excluded them.

### 2.3 Ethical considerations

The Research Ethics Review Committee of the Mongolian National University of Medical Sciences approved the study on 12 June 2025 (Approval No. 24–25/10–01). Participants received information about the purpose, voluntary nature, confidentiality, and anonymity of participation. Written informed consent was obtained before data collection. For participants aged <18 years, written guardian consent and participant assent were obtained in accordance with the approved protocol. For correctional participants, the consent process emphasized that participation or refusal would not affect legal status, correctional treatment, or access to services.

### 2.4 Measures

Drug-related problem severity was assessed using the 28-item Drug Abuse Screening Test (DAST-28). Scores range from 0 to 28, with higher scores indicating more drug-related problems and consequences. The updated analysis dataset contained binary responses for all 28 numbered items. The stored total score agreed with the sum of all 28 items in 169 of 170 complete records. The exact administered item wording and translation history were not retained in the analysis file; therefore, we report numbered item statistics but do not assign item-specific content or culturally validated domains.

The continuous DAST-28 total score was the primary outcome. Ordered descriptive score bands were derived from the verified total: 0, 1–5, 6–10, 11–15, and 16–28. These bands were used for supportive ordinal analysis and were not interpreted as locally validated clinical categories. The originally used DAST-28 ≥ 12 outcome was retained only as a study-defined sensitivity threshold and was not treated as a validated diagnostic, treatment, or referral cutoff.

Sociodemographic variables included age, sex, educational attainment, and study setting. Education grade 9 or above was reproducibly derived from the detailed educational-level variable. Substance-use variables included duration in months, patient-reported gabapentin/”Ban” use, synthetic cannabinoid/”Drop” use, methamphetamine/ice use, and public-place use. Polysubstance use was defined as use of at least two of gabapentin, synthetic cannabinoids, and methamphetamine/ice. Local drug names were treated as patient-reported exposures because toxicological confirmation was unavailable.

Behavioral variables included any syringe use, repeated self-use, giving a used syringe to others, using a syringe after another person, sharing syringes with others, disinfection before sharing, single-use non-shared syringe use, and injection of named substances. Variable-specific denominators were retained because several questions were conditional or had missing responses.

### 2.5 Data management and quality control

The original and additional datasets were merged one-to-one by participant identifier; all 181 identifiers matched without duplicates or shared-field mismatches. Labels and coding were reviewed, missingness was quantified, and the corrected duration-in-months field was used. Three stored score-band values conflicted with the stored total score, so ordered bands were derived directly from the total. One complete item record differed from the stored total by one point; the stored total was retained in the main analysis and the record was excluded in sensitivity analysis. Two participants had missing total scores. No values were imputed.

### 2.6 Statistical analysis

Continuous variables were summarized using mean and standard deviation and median with interquartile range. Categorical variables were summarized as n/N (%). Between-setting comparisons used Mann-Whitney *U* tests for continuous variables and chi-square or Fisher exact tests for categorical variables.

Internal consistency was evaluated among participants with complete responses to all 28 items. Cronbach's alpha was calculated with a 2000-resample percentile bootstrap confidence interval. Item endorsement, corrected item-total correlations, and alpha if each item was deleted were reported. For dichotomous items, alpha is equivalent to the Kuder-Richardson 20 coefficient. These analyses assess internal consistency, not dimensionality, cultural validity, or diagnostic validity.

The primary multivariable analysis used ordinary least-squares regression with HC3 heteroskedasticity-robust standard errors for the continuous DAST-28 score. Ordinary least squares was retained because its coefficients directly estimate adjusted mean-score differences; HC3 robust standard errors reduce reliance on homoskedastic residuals, and ordinal analyses assessed robustness to the outcome scale. Seven covariates were selected for the revised analysis on clinical and methodological grounds, without automated significance-based selection: age, female sex, education grade 9 or above, correctional versus psychiatric setting, duration of use per year, polysubstance use, and any syringe use. Variance inflation factors assessed multicollinearity.

Supportive analysis used proportional-odds ordinal logistic regression for the ordered score bands. A likelihood-ratio comparison with a less constrained multinomial model was used as an approximate global assessment of the proportional-odds restriction. Sensitivity analyses included logistic regression for DAST-28 ≥ 12 with the same seven covariates, Firth penalized logistic regression, alternate thresholds of ≥11 and ≥ 16, and analyses excluding participants aged <18 years, the one score-discrepant record, and participants without complete 28-item responses. Logistic discrimination, calibration, and optimism were summarized using AUC, Brier score, Hosmer-Lemeshow testing, Nagelkerke R^2^, and 1000-resample bootstrap internal validation. Analyses were explanatory; no model was presented as an externally validated prediction tool. Two-sided *p* < 0.05 was used.

ChatGPT (OpenAI; accessed July–August, 2026) was used to assist with drafting analysis code for implementation of the specified statistical workflow. The authors reviewed the code, executed the analyses, verified all numerical results against the retained dataset and output workbook, and retained responsibility for analytical decisions and interpretation.

## 3 Results

### 3.1 Participant characteristics

The study included 181 participants, of whom 76 (42.0%) were recruited from the psychiatric setting and 105 (58.0%) from correctional facilities. The median age was 26.0 years (IQR 22.0–29.0), 131/181 (72.4%) participants were male, and 162/181 (89.5%) had completed grade 9 or above. Psychiatric participants were younger than correctional participants (median 23.0 vs. 27.0 years, *p* < 0.001). Duration of substance use did not differ significantly between settings (median 34.5 vs. 36.0 months, *p* = 0.552). Participant characteristics are shown in Table 1.

**Table 1:** Participant characteristics by institutional setting.

Characteristic	Overall	Psychiatric	Correctional	*p*-value
Age, years	26.0 (22.0–29.0)	23.0 (20.0–27.0)	27.0 (24.0–31.0)	<0.001
Duration of substance use, months	36.0 (12.0–72.0)	34.5 (12.0–70.5)	36.0 (12.0–76.0)	0.552
DAST-28 total score	11.0 (6.0–16.0)	14.0 (10.0–17.0)	9.0 (4.0–14.0)	<0.001
Male sex	131/181 (72.4)	49/76 (64.5)	82/105 (78.1)	0.043
Education grade 9 or above	162/181 (89.5)	70/76 (92.1)	92/105 (87.6)	0.331
Gabapentin/”Ban” use	73/181 (40.3)	44/76 (57.9)	29/105 (27.6)	<0.001
Synthetic cannabinoid/”Drop” use	93/181 (51.4)	51/76 (67.1)	42/105 (40.0)	<0.001
Methamphetamine/ice use	146/181 (80.7)	62/76 (81.6)	84/105 (80.0)	0.791
Polysubstance use	100/181 (55.2)	54/76 (71.1)	46/105 (43.8)	<0.001
Public-place substance use	62/159 (39.0)	32/67 (47.8)	30/92 (32.6)	0.053
Any syringe use	81/181 (44.8)	50/76 (65.8)	31/105 (29.5)	<0.001

Note. Continuous values are median (IQR); categorical values are n/N (%). Variable-specific denominators are shown. Between-setting *p*-values use Mann-Whitney U, chi-square, or Fisher exact tests, as appropriate.

### 3.2 DAST-28 distribution and internal consistency

**Fig. 1:**
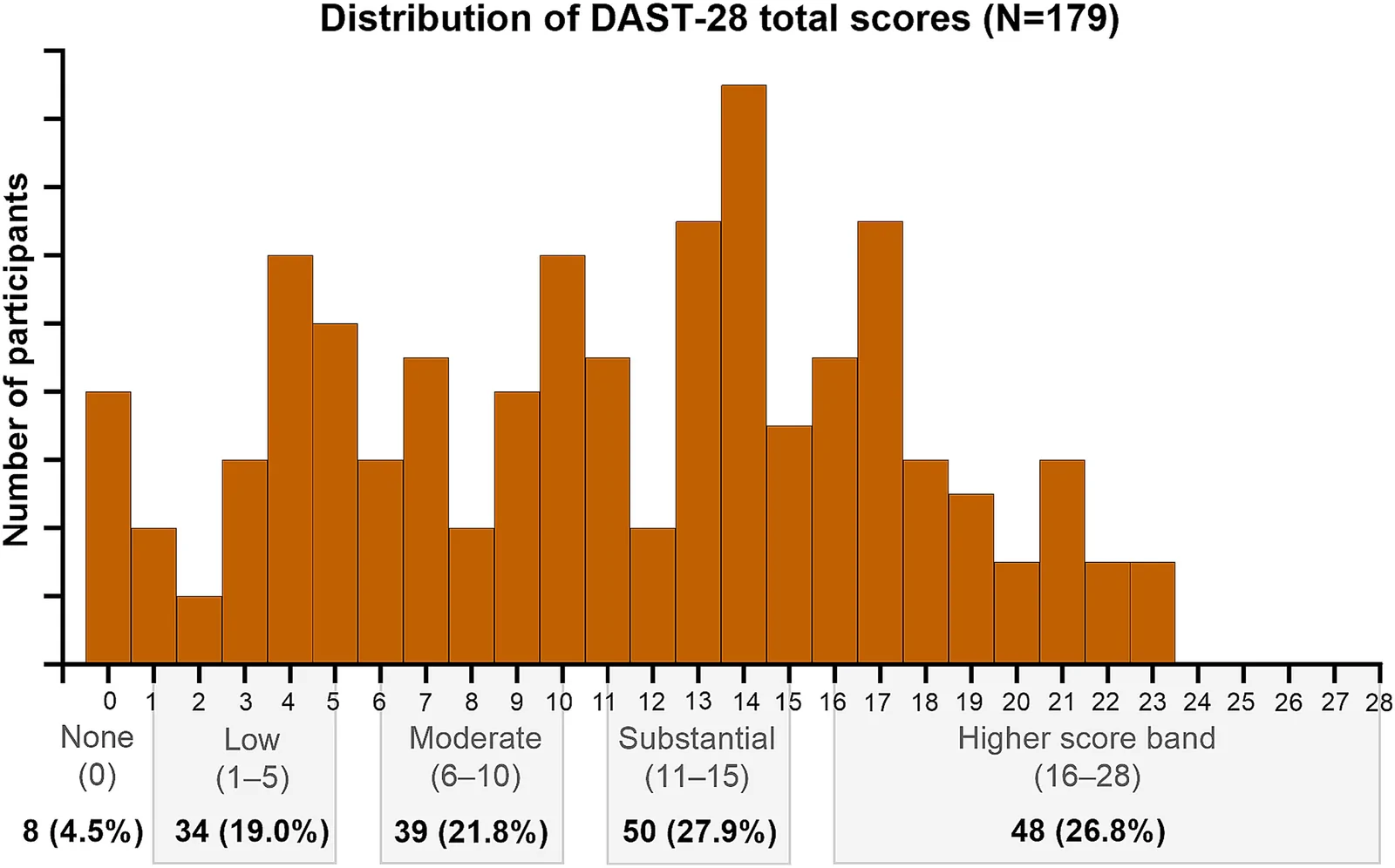
Distribution of DAST-28 total scores. Bars show participant counts for each observed total score among 179 participants with valid scores. Score-band labels are descriptive; no diagnostic threshold is implied.

Valid DAST-28 total scores were available for 179 participants. The mean was 11.15 (SD 6.04), the median was 11.0 (IQR 6.0–16.0), and the observed range was 0–23. Ordered descriptive score bands were: 0 in 8/179 (4.5%), 1–5 in 34/179 (19.0%), 6–10 in 39/179 (21.8%), 11–15 in 50/179 (27.9%), and 16–28 in 48/179 (26.8%). Scores were higher in the psychiatric setting than in the correctional setting (median 14.0 vs. 9.0, p < 0.001). The score distribution is shown in Fig. 1.

Among 170 participants with complete item responses, Cronbach's alpha was 0.882 (95% bootstrap CI 0.854–0.902); standardized alpha was 0.872. Alpha was 0.832 in psychiatric participants (*n* = 69) and 0.889 in correctional participants (*n* = 101). Across available item responses, endorsement ranged from 9.5% to 77.1%, corrected item-total correlations from 0.027 to 0.646, and alpha if an item was deleted from 0.873 to 0.885. No single item deletion materially improved reliability. Item-level findings are provided in Supplementary Table S1. The DAST-28 score-band distribution and internal-consistency summary are presented in Table 2.

**Table 2:** DAST-28 score-band distribution and internal consistency.

Measure / score band	Score range / basis	n or statistic	Percent / estimate
None	0	8	4.5
Low	1–5	34	19.0
Moderate	6–10	39	21.8
Substantial	11–15	50	27.9
Higher score band	16–28	48	26.8
Internal consistency	28 items; complete *N* = 170	Alpha	0.882 (95% CI 0.854–0.902)

Note. Score-band percentages use 179 participants with valid total scores. Internal consistency uses 170 participants with complete responses to all 28 items. The 95% CI for Cronbach's alpha is based on 2000 bootstrap resamples. Score bands are descriptive and are not locally validated diagnostic categories.

### 3.3 Primary multivariable analysis of continuous severity

**Fig. 2:**
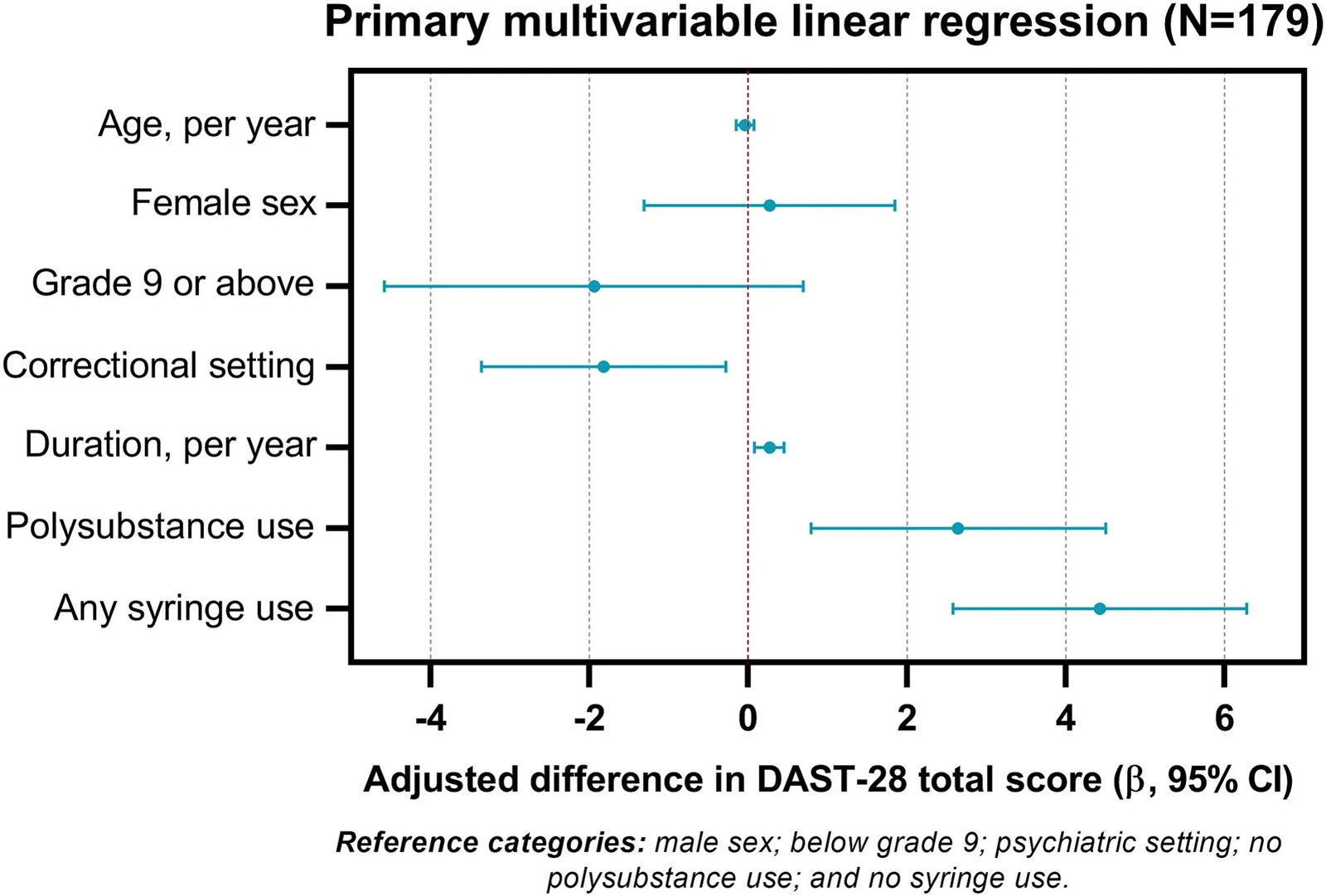
Adjusted associations with continuous DAST-28 total score. Points show adjusted regression coefficients and horizontal lines show 95% confidence intervals from the primary model with HC3 robust standard errors (*N* = 179). The vertical line indicates no adjusted score difference.

The primary model included 179 participants and explained 41.8% of score variance (adjusted R^2^ = 0.394). Correctional setting was associated with a 1.82-point lower DAST-28 score than psychiatric setting (95% CI -3.36 to −0.28; *p* = 0.020). Each additional year of use was associated with a 0.27-point higher score (95% CI 0.08–0.45; p = 0.005). Polysubstance use was associated with a 2.64-point higher score (95% CI 0.79–4.50; *p* = 0.005), and any syringe use with a 4.43-point higher score (95% CI 2.58–6.28; *p* < 0.001). VIFs ranged from 1.05 to 1.59, indicating no problematic multicollinearity in the final model. Adjusted estimates are displayed in Fig. 2. The corresponding regression coefficients are reported in Table 3.

**Table 3:** Primary multivariable linear regression for DAST-28 total score.

Predictor	Adjusted B	95% CI	p-value
Age, per year	−0.04	−0.15 to 0.07	0.464
Female sex	0.27	−1.31 to 1.85	0.736
Grade 9 or above	−1.94	−4.58 to 0.69	0.148
Correctional setting	−1.82	−3.36 to −0.28	0.020
Duration, per year	0.27	0.08 to 0.45	0.005
Polysubstance use	2.64	0.79 to 4.50	0.005
Any syringe use	4.43	2.58 to 6.28	<0.001

Note. B represents the adjusted mean difference in DAST-28 total score. Reference categories: male sex, below grade 9, psychiatric setting, no polysubstance use, and no syringe use. HC3 heteroskedasticity-robust standard errors were used.

**Fig. 3:**
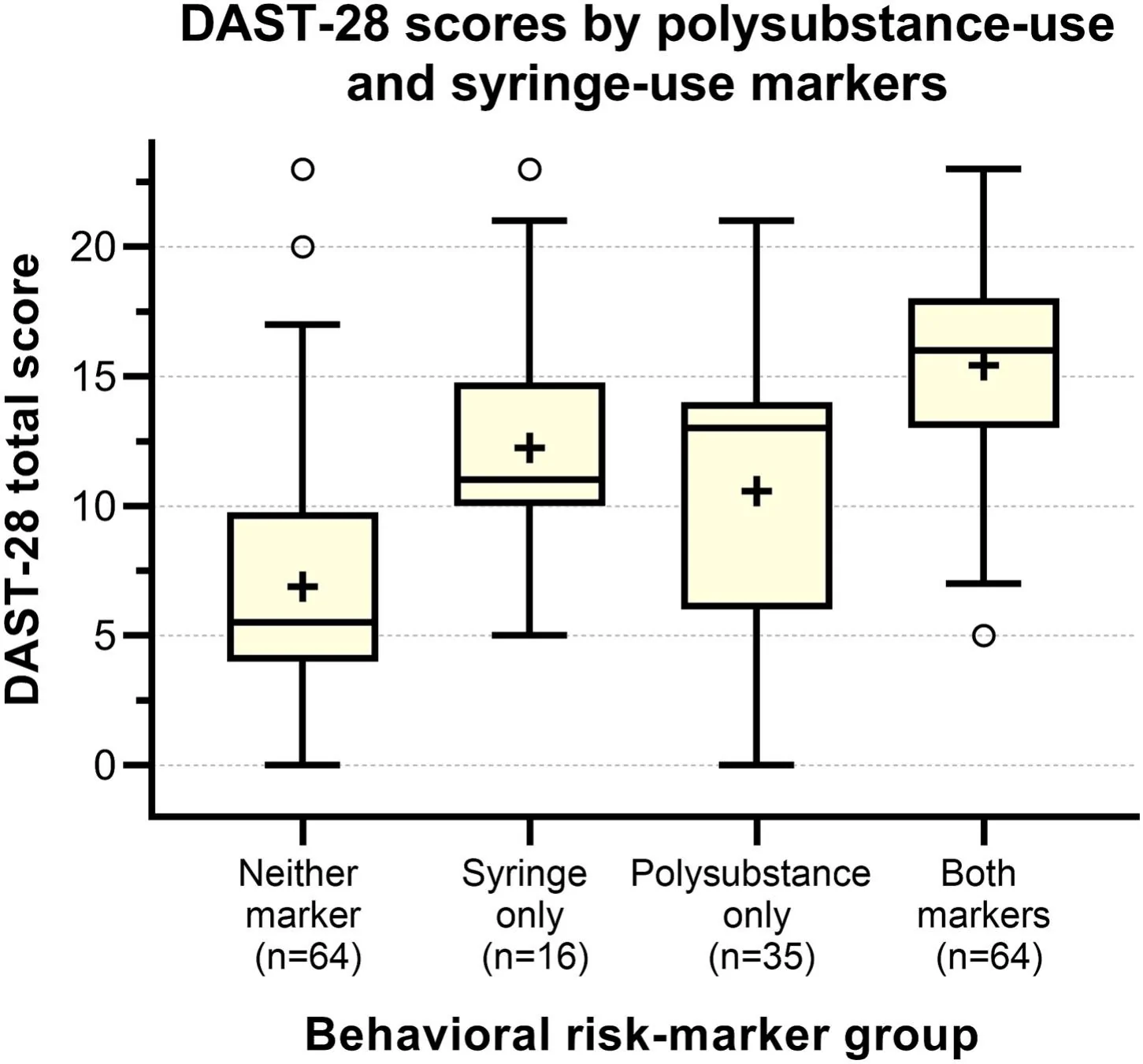
DAST-28 scores by combined polysubstance-use and syringe-use markers. Mutually exclusive groups were neither marker (*n* = 64), syringe use only (*n* = 16), polysubstance use only (*n* = 35), and both markers (n = 64). Boxes show interquartile ranges, horizontal lines show medians, plus signs show means, whiskers extend to 1.5 interquartile ranges, and circles identify observations beyond the whiskers. This figure is descriptive; adjusted results are reported in Table 3.

In descriptive cross-classification, median DAST-28 scores were 5.5 among participants with neither marker (*n* = 64), 11.0 with syringe use only (*n* = 16), 13.0 with polysubstance use only (*n* = 35), and 16.0 with both markers (n = 64). These unadjusted distributions are shown in Fig. 3; adjusted associations are reported in Table 3.

### 3.4 Ordinal and threshold sensitivity analyses

Ordinal logistic regression showed a concordant pattern. Correctional setting was associated with lower odds of a higher score band (common OR 0.50, 95% CI 0.27–0.92; *p* = 0.026). Higher score bands were associated with duration per year (common OR 1.10, 95% CI 1.02–1.18; *p* = 0.013), polysubstance use (common OR 3.01, 95% CI 1.54–5.90; *p* = 0.001), and any syringe use (common OR 5.61, 95% CI 2.75–11.44; *p* < 0.001). An approximate likelihood-ratio comparison did not show strong evidence against the proportional-odds restriction (*p* = 0.067). Adjusted ordinal regression estimates are shown in Table 4.

**Table 4:** Supportive ordinal logistic regression for ordered DAST-28 score bands.

Predictor	Common OR	95% CI	p-value
Age, per year	0.99	0.95 to 1.03	0.580
Female sex	1.13	0.60 to 2.13	0.704
Grade 9 or above	0.52	0.20 to 1.36	0.181
Correctional setting	0.50	0.27 to 0.92	0.026
Duration, per year	1.10	1.02 to 1.18	0.013
Polysubstance use	3.01	1.54 to 5.90	0.001
Any syringe use	5.61	2.75 to 11.44	<0.001

The study-defined DAST-28 ≥ 12 threshold was met by 89/179 participants (49.7%). In the seven-variable logistic sensitivity model, duration per year (AOR 1.13, 95% CI 1.02–1.24), polysubstance use (AOR 4.25, 95% CI 1.90–9.49), and any syringe use (AOR 3.05, 95% CI 1.32–7.04) were associated with the outcome. The model had an apparent AUC of 0.833 and a bootstrap optimism-corrected AUC of 0.805. Detailed alternate-threshold results are provided in Supplementary Table S2, exclusion analyses in Supplementary Table S3, model diagnostics and VIFs in Supplementary Table S4, and Firth estimates in Supplementary Table S5. Results were directionally consistent across these analyses.

Note. An OR > 1 indicates greater odds of membership in a higher ordered score band. Reference categories are as in Table 3. Score bands are descriptive and not locally validated clinical categories.

### 3.5 Syringe use and injection-related harm

Any syringe use was reported by 81/181 participants (44.8%). Among participants with valid responses, 33/97 (34.0%) reported sharing syringes with others, 31/97 (32.0%) reported using a syringe after another person, and 18/96 (18.8%) reported repeated self-use. Any injected substance was reported by 71/152 (46.7%). These behaviors were generally more common in the psychiatric setting, whereas single-use non-shared syringe use was less common (Table 5). Percentages represent this institutional sample and should not be interpreted as community prevalence.

**Table 5:** Syringe and injection-related behaviors by institutional setting.

Variable	Overall	Psychiatric	Correctional	p-value
Any syringe use	81/181 (44.8)	50/76 (65.8)	31/105 (29.5)	<0.001
Shares syringes with others	33/97 (34.0)	26/57 (45.6)	7/40 (17.5)	0.004
Repeated self-use of syringe	18/96 (18.8)	12/56 (21.4)	6/40 (15.0)	0.426
Gives own used syringe to others	14/97 (14.4)	11/57 (19.3)	3/40 (7.5)	0.144
Uses syringe after another person	31/97 (32.0)	25/57 (43.9)	6/40 (15.0)	0.003
Disinfects syringe before sharing	48/75 (64.0)	30/46 (65.2)	18/29 (62.1)	0.782
Single-use, non-shared syringe	43/96 (44.8)	16/56 (28.6)	27/40 (67.5)	<0.001
Any injected substance	71/152 (46.7)	49/64 (76.6)	22/88 (25.0)	<0.001
Injected gabapentin	56/152 (36.8)	43/64 (67.2)	13/88 (14.8)	<0.001
Injected amphetamine	33/152 (21.7)	25/64 (39.1)	8/88 (9.1)	<0.001

Note. Values are n/N (%); denominators vary because questions were conditional or missing. Between-setting p-values use chi-square or Fisher exact tests, as appropriate. Psychiatric and correctional percentages are not population prevalence estimates.

## 4 Discussion

### 4.1 Principal findings

This multicenter cross-sectional study provides an institutional profile of DAST-28 scores and injection-related risk in Mongolia. The revised analysis adds sample-specific internal-consistency evidence and no longer relies on an unvalidated binary cutoff as the primary outcome. Internal consistency was good, and the principal associations converged across continuous, ordered, and threshold analyses. Polysubstance use, syringe use, and longer duration of use were associated with higher DAST-28 scores; correctional setting was associated with lower continuous scores and lower ordered score bands after adjustment.

The magnitude of the syringe-use association was substantial at the group level: participants reporting any syringe use had, on average, an adjusted DAST-28 score 4.43 points higher than participants not reporting syringe use. This does not establish temporal direction or a minimal clinically important difference. Greater drug-related problem severity may increase the likelihood of syringe use, syringe use may mark more intensive patterns of use, or both may reflect shared underlying vulnerability.

### 4.2 Interpretation of the DAST-28 threshold

The originally used ≥12 threshold has no independently established diagnostic or treatment meaning in this Mongolian institutional population. It should therefore not be interpreted as a clinical decision boundary. Its role in this study is limited to a sensitivity analysis identifying a higher-score subgroup. The clinical and public-health relevance lies in the graded pattern across the full DAST-28 distribution and the convergence of associations across continuous, ordered, and alternate-threshold models. A local validation study with an external diagnostic criterion would be required before recommending a clinical cutoff.

### 4.3 Institutional context and generalizability

Psychiatric participants had higher unadjusted scores and more frequent injection-related behaviors than correctional participants. Psychiatric services may encounter individuals during acute addiction or mental-health crises, whereas correctional contact may reflect legal exposure as well as clinical severity (Drake et al., 2004). The adjusted setting difference was smaller than the unadjusted difference, suggesting that measured demographic and behavioral characteristics accounted for part of the institutional contrast. Nevertheless, the non-probability sample was recruited from highly specific services and cannot be extrapolated to all people who use drugs in Mongolia or to community prevalence.

### 4.4 Injection-related harm and patient-reported substances

Syringe use, sharing, and use after another person indicate potential exposure to blood-borne viruses and other injection-related harms. Sterile syringe access, safer-use counseling, testing and treatment for HIV and viral hepatitis, overdose prevention, and linkage to addiction treatment are established components of harm reduction (Platt et al., 2017; World Health Organization, 2026; World Health Organization, United Nations Office on Drugs and Crime, and UNAIDS, 2012). The present findings may inform focused questions and referral pathways within psychiatric and correctional services, but this study did not test the effectiveness of any intervention.

Patient-reported gabapentin/”Ban” and synthetic cannabinoid/”Drop” use were common and clustered with higher severity in unadjusted analyses. International evidence documents gabapentinoid misuse and diversion (Bonnet & Scherbaum, 2017; Evoy et al., 2017) and severe harms associated with synthetic cannabinoids (Castaneto et al., 2014; Tait et al., 2016). Because local names were not chemically verified, they should be interpreted as patient-reported risk signals rather than confirmed pharmacological categories.

### 4.5 Implications for assessment and future evaluation

**Fig. 4:**
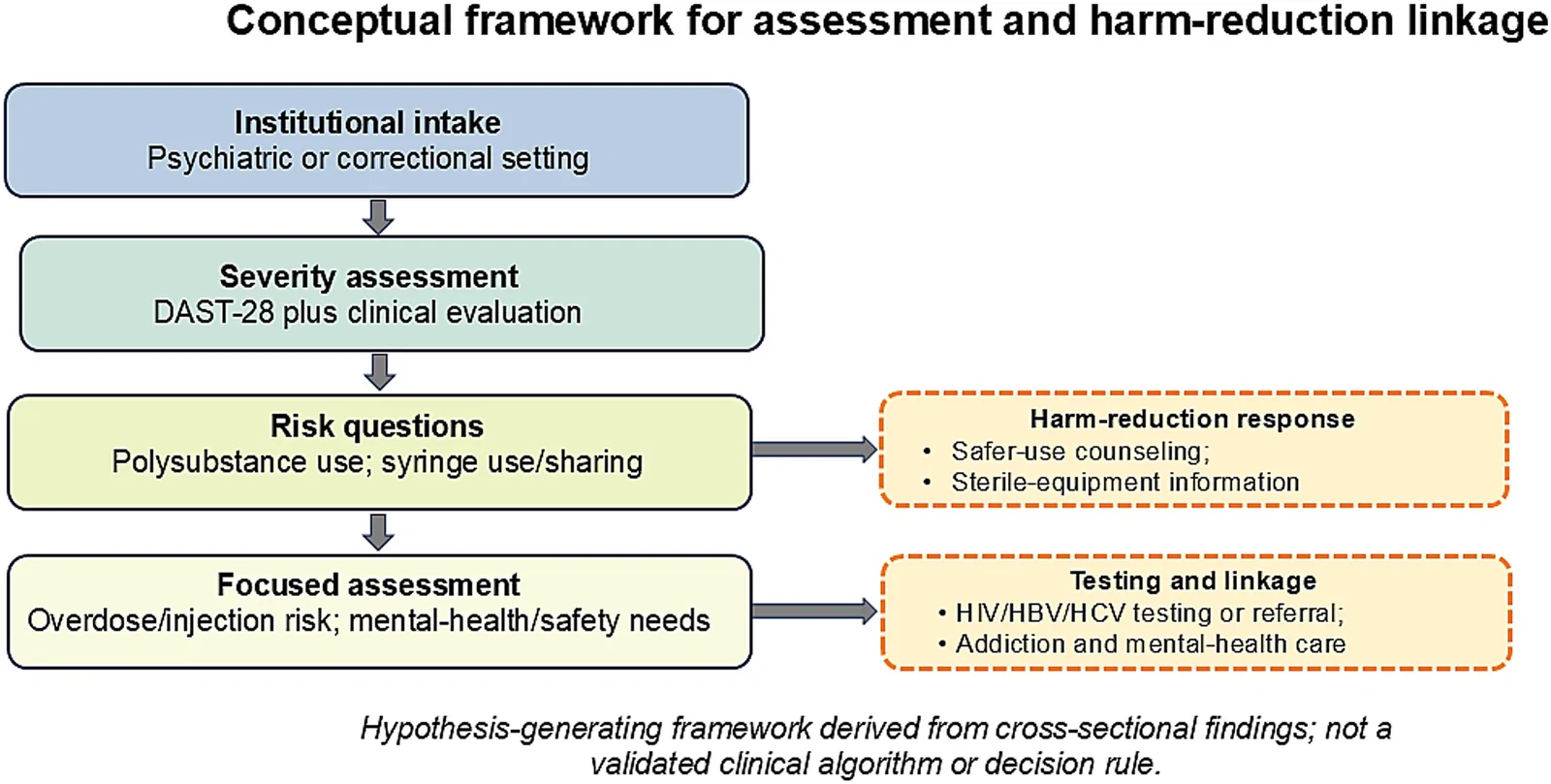
Conceptual framework for assessment and harm-reduction linkage in psychiatric and correctional settings. This framework is hypothesis-generating, was not prospectively validated, and is not a clinical decision rule or treatment algorithm.

Within psychiatric and correctional services, DAST-28 scores may be considered alongside clinical evaluation rather than used as a stand-alone diagnosis. Direct questions about syringe use, sharing, reuse, disposal, and use after another person may identify needs not captured by a total score alone. Reports of syringe use or injection may prompt consideration of HIV, HBV, and HCV testing and referral, subject to local protocols and resources. Any institutional pathway should be prospectively evaluated before being treated as a validated clinical algorithm. A hypothesis-generating framework is shown in Fig. 4.

### 4.6 Strengths and limitations

Strengths include recruitment from two high-relevance institutional settings, use of all 28 item responses to verify scoring, sample-specific internal-consistency analysis, explicit correction of score-band coding, a clinically specified parsimonious model, low VIFs, bootstrap internal validation, and convergence across multiple outcome parameterizations.

Several limitations remain. The cross-sectional design precludes causal and temporal inference. The institutional convenience sample limits generalizability. Substance use and syringe behaviors were self-reported and may be affected by recall, stigma, or legal concerns. Toxicological confirmation and biological testing were unavailable. The exact administered wording and translation history of the 28 items were not retained in the analysis file, so internal consistency could be assessed but item content, dimensionality, measurement invariance, and cultural validity could not be fully evaluated. One complete record had a one-point discrepancy between the item sum and stored total; exclusion did not materially change findings. Several syringe variables had conditional or missing responses. Finally, model performance was internally assessed only and should not be interpreted as external predictive validity.

## 5 Conclusions

DAST-28 showed good internal consistency in this institutional sample. Higher DAST-28 scores were associated with polysubstance use, any syringe use, and longer duration of use. These associations were consistent across continuous, ordered, and threshold analyses. The findings may inform comprehensive assessment, direct syringe-risk questions, and referral for harm-reduction and blood-borne virus services within psychiatric and correctional settings. They do not establish causality, a validated clinical cutoff, intervention effectiveness, or prevalence beyond the study sample.

## Declaration of generative AI and AI-assisted technologies in the manuscript preparation process

During preparation of this revised work, the authors used ChatGPT (OpenAI; accessed July–August, 2026) to assist with language editing, manuscript organization, consistency checking, and statistical code drafting. After using this tool, the authors reviewed and edited the content, reviewed and executed the analytical code, independently verified all numerical results against the retained dataset and analysis outputs, and take full responsibility for the content of the publication.

## CRediT authorship contribution statement

**Bilguun Nyamdorj:** Writing – original draft, Methodology, Investigation, Data curation, Conceptualization. **Khongorzul Davaadorj:** Validation, Resources, Investigation, Data curation. **Tergel Khuyag:** Validation, Resources, Investigation, Data curation. **Uranchimeg Mungunkhuyag:** Writing – original draft, Resources, Investigation, Data curation. **Munkhnaran Mandakh:** Writing – original draft, Resources, Investigation, Data curation. **Davaalkham Jagdagsuren:** Writing – original draft, Validation, Resources, Data curation. **Nursabi Khizatkhan:** Writing – original draft, Visualization, Validation. **Khishigsuren Zuunnast:** Writing – review & editing, Validation, Supervision, Project administration, Investigation, Data curation. **Dariimaa Ganbat:** Writing – review & editing, Visualization, Validation, Methodology, Formal analysis. **Oyunsuren Davaasuren:** Writing – review & editing, Validation, Supervision, Project administration, Methodology, Conceptualization.

## Consent for publication

Not applicable; no individually identifiable participant information is reported.

## Ethics approval and consent to participate

The Research Ethics Review Committee of the Mongolian National University of Medical Sciences approved the study on 12 June 2025 (Approval No. 24–25/10–01). Written informed consent was obtained. For participants aged <18 years, guardian consent and participant assent were obtained. For correctional participants, participation or refusal did not affect legal status, treatment, or access to services.

## Ethics declaration

Written informed consent to take part in the study and to publish the article has been obtained from all participants or their legal representatives. The privacy rights of participants have been observed.

This study was performed in compliance with relevant laws, regulatory frameworks and guidelines where the research took place.

This study was approved by the Research Ethics Review Committee of the Mongolian National University of Medical Sciences; approved 12 June 2025; (Approval No. Approval No. 24–25/10–01.)

## Funding

This research did not receive any specific grant from funding agencies in the public, commercial, or not-for-profit sectors.

## Declaration of competing interest

The authors declare that they have no known competing financial interests or personal relationships that could have appeared to influence the work reported in this paper.

## Data Availability

Individual-level data contain sensitive psychiatric, substance-use, and correctional information. Public repository deposition is not permitted because of re-identification risk. De-identified data may be made available by the corresponding author upon reasonable request and subject to institutional and ethics approval.
