# Supplementary material for: DAST-28 severity and injection-related harm risks among people using psychoactive substances in psychiatric and correctional settings in Mongolia: a multicenter cross-sectional study
Source: Addict Behav Rep. 2026 Aug 20;24:100736. doi: 10.1016/j.abrep.2026.100736 (PMC13524739; doi:10.1016/j.abrep.2026.100736)
Supplement: Supplementary file 1 [file mmc1.docx]

Supplementary Material

DAST-28 Severity and Injection-Related Harm Risks Among People Using Psychoactive Substances in Psychiatric and Correctional Settings in Mongolia: A Multicenter Cross-Sectional Study

# Supplementary Table S1. DAST-28 item-level endorsement and internal-consistency diagnostics

Exact administered item wording and translation history were not retained in the analysis file. Items are therefore identified only by number, and no item-specific clinical interpretation is made. Overall Cronbach's alpha=0.882 (95% bootstrap CI 0.854-0.902); complete-item N=170.

| **Item** | **Valid N** | **Endorsed n** | **Endorsed %** | **Corrected item-total r** | **Alpha if deleted** |
| --- | --- | --- | --- | --- | --- |
| Item 1 | 179 | 60 | 33.5 | 0.552 | 0.875 |
| Item 2 | 179 | 76 | 42.5 | 0.643 | 0.873 |
| Item 3 | 177 | 70 | 39.5 | 0.560 | 0.875 |
| Item 4 | 179 | 27 | 15.1 | 0.176 | 0.883 |
| Item 5 | 178 | 33 | 18.5 | 0.321 | 0.880 |
| Item 6 | 178 | 17 | 9.6 | 0.129 | 0.883 |
| Item 7 | 179 | 20 | 11.2 | 0.027 | 0.885 |
| Item 8 | 179 | 80 | 44.7 | 0.530 | 0.876 |
| Item 9 | 179 | 132 | 73.7 | 0.325 | 0.881 |
| Item 10 | 179 | 106 | 59.2 | 0.458 | 0.877 |
| Item 11 | 179 | 138 | 77.1 | 0.491 | 0.877 |
| Item 12 | 179 | 115 | 64.2 | 0.472 | 0.877 |
| Item 13 | 178 | 137 | 77.0 | 0.428 | 0.878 |
| Item 14 | 179 | 108 | 60.3 | 0.601 | 0.874 |
| Item 15 | 179 | 66 | 36.9 | 0.467 | 0.877 |
| Item 16 | 179 | 88 | 49.2 | 0.603 | 0.874 |
| Item 17 | 179 | 79 | 44.1 | 0.514 | 0.876 |
| Item 18 | 179 | 32 | 17.9 | 0.339 | 0.880 |
| Item 19 | 179 | 51 | 28.5 | 0.416 | 0.878 |
| Item 20 | 179 | 17 | 9.5 | 0.244 | 0.882 |
| Item 21 | 179 | 74 | 41.3 | 0.466 | 0.877 |
| Item 22 | 179 | 53 | 29.6 | 0.187 | 0.884 |
| Item 23 | 178 | 113 | 63.5 | 0.646 | 0.873 |
| Item 24 | 179 | 103 | 57.5 | 0.625 | 0.873 |
| Item 25 | 178 | 81 | 45.5 | 0.491 | 0.877 |
| Item 26 | 179 | 65 | 36.3 | 0.458 | 0.877 |
| Item 27 | 179 | 35 | 19.6 | 0.271 | 0.882 |
| Item 28 | 177 | 18 | 10.2 | 0.237 | 0.882 |

Note. Endorsement percentages use each item's available denominator. Corrected item-total correlations and alpha-if-deleted estimates use complete-item records.

# Supplementary Table S2. Logistic sensitivity analyses at alternate DAST-28 thresholds

| **Threshold** | **Events** | **N** | **Predictor** | | **Adjusted OR** | **95% CI** | **p-value** |
| --- | --- | --- | --- | --- | --- | --- | --- |
| ≥11 | 98 | 179 | | Duration, per year | 1.12 | 1.02-1.24 | 0.017 |
| ≥11 | 98 | 179 | | Polysubstance use | 3.33 | 1.50-7.41 | 0.003 |
| ≥11 | 98 | 179 | | Any syringe use | 4.62 | 1.96-10.90 | <0.001 |
| ≥12 | 89 | 179 | | Duration, per year | 1.13 | 1.02-1.24 | 0.015 |
| ≥12 | 89 | 179 | | Polysubstance use | 4.25 | 1.90-9.49 | <0.001 |
| ≥12 | 89 | 179 | | Any syringe use | 3.05 | 1.32-7.04 | 0.009 |
| ≥16 | 48 | 179 | | Duration, per year | 1.06 | 0.94-1.20 | 0.310 |
| ≥16 | 48 | 179 | | Polysubstance use | 3.46 | 1.19-10.04 | 0.022 |
| ≥16 | 48 | 179 | | Any syringe use | 3.57 | 1.34-9.54 | 0.011 |

Note. Every model included age, female sex, education grade 9 or above, correctional setting, duration per year, polysubstance use, and any syringe use. Thresholds are analytical and not validated clinical cutoffs.

# Supplementary Table S3. Exclusion sensitivity analyses

| **Analysis** | **N** | **Outcome** | **Predictor** | **Estimate** | **95% CI** | **p-value** |
| --- | --- | --- | --- | --- | --- | --- |
| Exclude age <18 | 177 | Continuous | Duration/year | 0.26 | 0.08-0.45 | 0.005 |
| Exclude age <18 | 177 | Continuous | Polysubstance | 2.69 | 0.83-4.55 | 0.005 |
| Exclude age <18 | 177 | Continuous | Syringe use | 4.45 | 2.59-6.30 | <0.001 |
| Exclude age <18 | 177 | ≥12 logistic | Duration/year | 1.12 | 1.02-1.24 | 0.017 |
| Exclude age <18 | 177 | ≥12 logistic | Polysubstance | 4.37 | 1.95-9.80 | <0.001 |
| Exclude age <18 | 177 | ≥12 logistic | Syringe use | 3.07 | 1.33-7.11 | 0.009 |
| Exclude ID 171 | 178 | Continuous | Duration/year | 0.27 | 0.09-0.46 | 0.004 |
| Exclude ID 171 | 178 | Continuous | Polysubstance | 2.55 | 0.70-4.40 | 0.007 |
| Exclude ID 171 | 178 | Continuous | Syringe use | 4.31 | 2.46-6.16 | <0.001 |
| Complete items only | 170 | Continuous | Duration/year | 0.27 | 0.08-0.46 | 0.005 |
| Complete items only | 170 | Continuous | Polysubstance | 2.52 | 0.56-4.47 | 0.012 |
| Complete items only | 170 | Continuous | Syringe use | 4.92 | 2.99-6.85 | <0.001 |
| Complete items only | 170 | ≥12 logistic | Duration/year | 1.13 | 1.02-1.24 | 0.014 |
| Complete items only | 170 | ≥12 logistic | Polysubstance | 3.83 | 1.65-8.89 | 0.002 |
| Complete items only | 170 | ≥12 logistic | Syringe use | 4.09 | 1.70-9.83 | 0.002 |

Note. Continuous-model estimates are adjusted mean-score differences; logistic estimates are adjusted odds ratios. All models used the same seven covariates as the primary analysis.

# Supplementary Table S4. Model diagnostics, internal validation, and multicollinearity

| **Diagnostic** | **Value** |
| --- | --- |
| Primary continuous-model N | 179 |
| Primary model R^2^ | 0.418 |
| Primary adjusted R^2^ | 0.394 |
| Logistic sensitivity N | 179 |
| DAST-28 ≥12 events / non-events | 89 / 90 |
| Apparent AUC | 0.833 |
| Bootstrap-corrected AUC | 0.805 |
| Brier score | 0.168 |
| Nagelkerke R^2^ | 0.417 |
| Hosmer-Lemeshow χ² (df=8), p | 8.843; p=0.356 |
| Mean bootstrap calibration slope | 0.851 |
| Median bootstrap calibration slope | 0.843 |
| Approximate proportional-odds LR p | 0.067 |

Note. Bootstrap estimates used 1,000 resamples. Logistic model performance is internally assessed and is not external predictive validation.

**Variance inflation factors.**

| **Predictor** | **VIF** |
| --- | --- |
| Age, per year | 1.39 |
| Female sex | 1.07 |
| Grade 9 or above | 1.05 |
| Correctional setting | 1.25 |
| Duration, per year | 1.21 |
| Polysubstance use | 1.47 |
| Any syringe use | 1.59 |

Note. VIF values do not indicate problematic multicollinearity.

# Supplementary Table S5. Firth penalized logistic sensitivity model for DAST-28 ≥12

| **Predictor** | **Firth OR** | **Approximate 95% CI** | **p-value** |
| --- | --- | --- | --- |
| Age, per year | 0.97 | 0.91-1.03 | 0.304 |
| Female sex | 0.87 | 0.38-2.01 | 0.750 |
| Grade 9 or above | 0.52 | 0.15-1.77 | 0.296 |
| Correctional setting | 0.61 | 0.28-1.32 | 0.208 |
| Duration, per year | 1.12 | 1.02-1.23 | 0.022 |
| Polysubstance use | 3.99 | 1.80-8.81 | <0.001 |
| Any syringe use | 2.92 | 1.28-6.69 | 0.011 |

Note. Firth bias-reduced logistic regression used the same seven covariates as the conventional logistic model. Confidence intervals and p-values are Wald approximations from the penalized information matrix.
