# Supplementary material for: DAST-28 severity and injection-related harm risks among people using psychoactive substances in psychiatric and correctional settings in Mongolia: a multicenter cross-sectional study
Source: Addict Behav Rep. 2026 Aug 20;24:100736. doi: 10.1016/j.abrep.2026.100736 (PMC13524739; doi:10.1016/j.abrep.2026.100736)
Supplement: Supplementary file 2 [file mmc2.docx]

STROBE Statement - Checklist for Cross-Sectional Studies

**Manuscript:** DAST-28 Severity and Injection-Related Harm Risks Among People Using Psychoactive Substances in Psychiatric and Correctional Settings in Mongolia: A Multicenter Cross-Sectional Study

| **Item** | **Recommendation** | **Reported in manuscript** |
| --- | --- | --- |
| 1a | Indicate the study design with a commonly used term in the title or abstract | Title; Abstract - Methods |
| 1b | Provide an informative and balanced summary of what was done and found | Abstract |
| 2 | Explain the scientific background and rationale | Introduction, paragraphs 1-3 |
| 3 | State specific objectives, including prespecified hypotheses | Introduction, final paragraph |
| 4 | Present key elements of study design early | Methods 2.1 |
| 5 | Describe setting, locations, and relevant dates | Methods 2.1-2.2 |
| 6a | Give eligibility criteria and sources/methods of participant selection | Methods 2.2 |
| 6b | For matched studies, give matching criteria | Not applicable |
| 7 | Clearly define outcomes, exposures, predictors, confounders, and effect modifiers | Methods 2.4 |
| 8 | Give sources of data and details of measurement methods | Methods 2.4-2.5 |
| 9 | Describe efforts to address potential sources of bias | Methods 2.5-2.6; Discussion 4.6 |
| 10 | Explain how study size was arrived at | Methods 2.2: all 181 eligible records |
| 11 | Explain how quantitative variables were handled | Methods 2.4 and 2.6 |
| 12a | Describe all statistical methods, including control of confounding | Methods 2.6 |
| 12b | Describe methods used to examine subgroups/interactions | Descriptive Fig. 3; no interaction model presented |
| 12c | Explain how missing data were addressed | Methods 2.5: variable-specific denominators; no imputation |
| 12d | Describe analytical methods taking account of sampling strategy | Not applicable; convenience institutional sample |
| 12e | Describe sensitivity analyses | Methods 2.6; Results 3.4; Supplementary Tables S2-S5 |
| 13a | Report numbers of individuals at each stage | Results 3.1; N=181, valid total N=179, complete items N=170 |
| 13b | Give reasons for non-participation at each stage | Not available/applicable to retained analysis records |
| 13c | Consider use of a flow diagram | Not required; no multistage exclusions |
| 14a | Give characteristics of study participants and information on exposures/confounders | Results 3.1; Table 1 |
| 14b | Indicate number with missing data for each variable | Tables 1 and 5; variable-specific denominators; Supplement |
| 14c | Summarize follow-up time | Not applicable to cross-sectional design |
| 15 | Report outcome events or summary measures | Results 3.2-3.5; Tables 2 and 5 |
| 16a | Give unadjusted and adjusted estimates and precision | Results 3.3-3.4; Tables 3-4; Supplement |
| 16b | Report category boundaries when continuous variables categorized | Methods 2.4; Table 2 |
| 16c | Translate relative risk into absolute risk when relevant | Not applicable |
| 17 | Report other analyses, including sensitivity analyses | Results 3.4; Supplementary Tables S2-S5 |
| 18 | Summarize key results with reference to objectives | Discussion 4.1 |
| 19 | Discuss limitations, direction and magnitude of potential bias | Discussion 4.6 |
| 20 | Give cautious overall interpretation | Discussion 4.1-4.5; Conclusions |
| 21 | Discuss generalizability | Discussion 4.3 and 4.6; Conclusions |
| 22 | Give source of funding and role of funders | Declarations: Funding |

This checklist is based on the STROBE cross-sectional reporting recommendations. Page and line numbers may change after Editorial Manager conversion; section and table locations are provided for stability.
